# On the effects of 30.5 GHz sinusoidal wave exposure on glioblastoma organoids

**DOI:** 10.3389/fonc.2024.1307516

**Published:** 2024-05-31

**Authors:** Elena Rampazzo, Luca Persano, Nissar Karim, George Hodgking, Rosanna Pinto, Arianna Casciati, Mirella Tanori, Alessandro Zambotti, Silvia Bresolin, Alice Cani, Alessandro Pannicelli, Ilan W. Davies, Cristopher Hancock, Cristiano Palego, Giampietro Viola, Mariateresa Mancuso, Caterina Merla

**Affiliations:** ^1^ Department of Women’s and Children’s Health (SDB), University of Padova, Padova, Italy; ^2^ Istituto di Ricerca Pediatrica Città della Speranza, Padova, Italy; ^3^ School of Computer Science and Engineering, Bangor University, Bangor, United Kingdom; ^4^ CREO Medical Limited, Bath, United Kingdom; ^5^ National Italian Agency for Energy New Technologies and Sustainable Economic Development (ENEA), Division of Health Protection Technologies, Rome, Italy; ^6^ Technical Unit of Energetic Efficiency, National Italian Agency for Energy New Technologies and Sustainable Economic Development (ENEA), Rome, Italy

**Keywords:** millimeter waves, numerical and experimental dosimetry, transcriptomics, glioblastoma organoids, combined treatments

## Abstract

**Introduction:**

Glioblastoma (grade IV) is the most aggressive primary brain tumor in adults, representing one of the biggest therapeutic challenges due to its highly aggressive nature. In this study, we investigated the impact of millimeter waves on tridimensional glioblastoma organoids derived directly from patient tumors. Our goal was to explore novel therapeutic possibilities in the fight against this challenging disease.

**Methods:**

The exposure setup was meticulously developed in-house, and we employed a comprehensive dosimetry approach, combining numerical and experimental methods. Biological endpoints included a global transcriptional profiling analysis to highlight possible deregulated pathways, analysis of cell morphological changes, and cell phenotypic characterization which are all important players in the control of glioblastoma progression.

**Results and discussion:**

Our results revealed a significant effect of continuous millimeter waves at 30.5 GHz on cell proliferation and apoptosis, although without affecting the differentiation status of glioblastoma cells composing the organoids. Excitingly, when applying a power level of 0.1 W (Root Mean Square), we discovered a remarkable (statistically significant) therapeutic effect when combined with the chemotherapeutic agent Temozolomide, leading to increased glioblastoma cell death. These findings present a promising interventional window for treating glioblastoma cells, harnessing the potential therapeutic benefits of 30.5 GHz CW exposure. Temperature increase during treatments was carefully monitored and simulated with a good agreement, demonstrating a negligible involvement of the temperature elevation for the observed effects. By exploring this innovative approach, we pave the way for improved future treatments of glioblastoma that has remained exceptionally challenging until now.

## 1 Introduction

Recently, a growing interest for applications of millimeter wave (MMW) signals (in the band from 30 to 300 GHz) in proximity of the human body has emerged (1–3). These frequencies are going to be used in new 5G/6G technologies to support IoT (4), fast big data transmission, and to possibly implement virtual reality in diverse sectors, from industry (5) to medical applications (6). MMW incident power density for these usages is low (order of few W/m^2^) usually not determining temperature increase in the exposed target (7).

Beyond such important use, MMW signals start to be also studied as a possible therapeutic tool to develop new ablative therapies in oncology (8) and, notably, to target even deep-seated organs trough endoscopic approaches (9). Indeed, MMW signals maximally limit the damage to healthy anatomical structures surrounding cancers due to their much higher spatial resolution, if compared to radiofrequencies and microwaves (10). In this latter application, the increase of temperature induced by the MMWs has to be high (incident power density in the order of thousands of W/m^2^) guarantying the targeted tissue necrosis (2).

**Figure 1:**
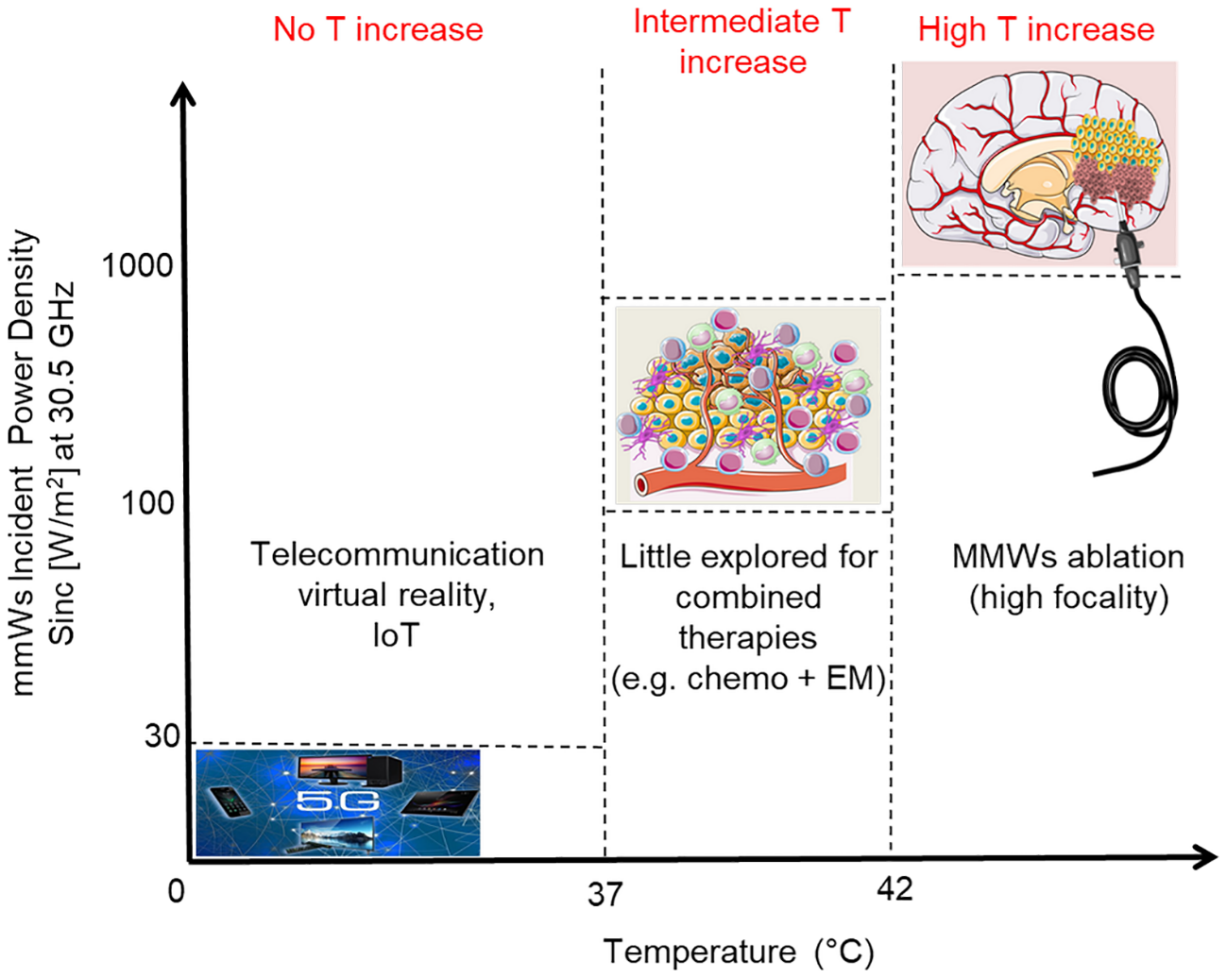
Scheme of applications of MMWs as a function of the Incident Power Density (order of magnitude); the induced temperature increases are also reported. Incident Power Density limit associated to 5G refers to ICNIRP reference levels for local exposure (over 6 minutes) of the General Public at 30.5 GHz (ICNIRP 2020).

Another possible option for the use of MMWs could be in a medium thermal regimen (increase of tissue temperature below the threshold for tissue necrosis) as adjuvant approach to the standard treatments for cancer (i.e. radiotherapy and chemotherapy) (11). This last option, very poorly investigated so far, has the rationale that a mild temperature increase can still limit damage to surrounding systems, nevertheless promoting alterations in the microenvironment of the tumor and in its phenotype. All these features could be exploited in order to potentiate the effects of standard therapies, with the final aim of eradicating the tumor. Moreover, cancer stem cells and their microenvironment could be affected as well (12, 13). A scheme of the mentioned electromagnetic (EM) regimen interactions with the tumor microenvironment and related applications/effects is reported in **Figure 1**.

In this paper, we characterized the bio-response of a relevant glioblastoma multiforme (GBM) model to a 30.5 GHz continuous wave (CW) signal tailored for localized non-ablative cancer treatments (the last-mentioned option or intermediate thermal regimen in **Figure 1**). The model was constituted by human patient-derived primary cells to mimic the intrinsic cellular heterogeneity that characterize such a deadly disease.

The frequency of 30.5 GHz was selected as a compromise between high spatial resolution and penetration depth of the electric field, covering several cell layers, while also considering the cost and the availability of high-power semiconductor technology for the generation and monitoring equipment. 30.5 GHz is within the Ka-band (26.5–40 GHz) of the millimeter-wave spectrum, which has been proposed for various 5G applications, such as wireless backhaul, fixed wireless access, and vehicular communications. This results in the availability of off-the-shelf millimeter-wave modules with increasing and cost-effective power handling capabilities. The wavelength at 30.5 GHz (< 1 cm) could potentially drive a new generation of Creo Medical systems for ablation of tumors with diameter in the millimeter-range with a more than two-fold resolution improvement over earlier 14.5 GHz systems (14). The move to higher frequency, and yet equally distant from the 22.5 GHz resonant frequency of water molecules, also sought to exploit strong water absorption and potentially achieve large penetration depth. Indeed, even the work at 14.5 GHz resulted in spherical ablation zones that were larger than expected from thermal conduction and the electric field penetration at these MMW frequencies. Therefore, the achievable ablation zone at the operation frequency enabled by the present technology became of interest. Furthermore, continuous wave millimeter-wave irradiation is increasingly being considered due to the potential to selectively elicit non-thermal protein dielectric alterations that are not consistent with the expected sample temperature increase (15).

High grade gliomas, including GBM, are the most frequent and aggressive primary malignant brain tumor in adults with a median overall survival of 17 months. The gold standard therapeutic protocol for GBM is a multimodal approach that combines surgery, radiotherapy, and adjuvant chemotherapy with temozolomide (TMZ), a DNA alkylating agent (16). Although the introduction of such a therapeutic protocol led to a significant improvement of patient survival (some months), it still remains disappointing (17). Indeed, the locally infiltrative nature of the tumor often prevents complete surgical resection and leads to inevitable early recurrences (18).

In order to develop more effective therapies, in the last decade, a broad investigation of genetic, molecular and cellular features of this tumor was pursued. GBM has an intrinsic intra-tumoral heterogeneity, also in terms of molecular subtypes, which may have a strong implication in the emergence of TMZ resistance. This point determines considerable variations between individual treatment responses (19). In order to take into account the intrinsic heterogeneous nature of GBM tumor mass, composed by undifferentiated and differentiated cells dynamically changing during time, a three dimensional (3D) model (patient-derived) named organoid was recently proposed (20). Organoids are cell assemblies built up starting from a small group of isolated cells able to grow and reproduce the structure and complexity of the real tumors. These 3D organized structures contain a nucleus of dispersed cells surrounded by a mixture of progenitor and stem-like cells together with specific sub-populations of differentiated cells mostly placed on the external organoid layers. Organoids generated from primary patient’s derived GMB cells are not only representative of the heterogeneity of the pathology in terms of cell composition, but also can reproduce inter-individual tumor heterogeneity (20).

The purpose of this study was threefold: it sought to 1) assess the threshold input power density for the occurrence of some stress/modifications upon suspended organoids; 2) experimentally assess possible effects of MMW signal in the medium thermal regimen investigated so far; 3) survey the occurrence of coupled non-thermal effects in such a frequency range. For this reason, in this study we compared different MMW exposure conditions, although maintaining the same delivered energy.

The followed exposure protocols took into account two conditions: one delivering twice as much power but over a half of the time than the other (i.e. 0.2 W root mean square RMS provided for 10 minutes and 0.1 W RMS provided for 20 minutes). These two conditions determine different temperature increases, directly dependent on the incident MMW power and inversely dependent on the exposure time, but keeping the same total delivered energy. The *in vitro* EM exposure was performed on a suitable biological holder using a horn antenna connected to a CW 30.5 GHz generator designed and developed by CREO Medical (UK). The exposure setup was completely characterized from a dosimetric point of view, both numerically and experimentally, to allow complete control of the provided EM doses and temperature elevation in our bio-samples. Temperature and ambient conditions were carefully controlled during the experiments to guarantee that EM exposure would not exceeding 37°C, a non-physiological condition for cells.

The investigated biological end points included: a global transcriptional profiling analysis of sham and exposed GBM cells, to highlight possible deregulated pathways; cell viability; analysis of cell morphological changes, cell phenotypic characterization and proliferation assessment, all important players in the control of GBM progression (18–20).

Globally, our investigation sheds light on the characterization of possible bio-effects of MMW (30.5 GHz CW) on GBM organoids, highlighting cell and molecular responses in a relevant 3D tumor model.

## 2 Materials and methods

### 2.1 Exposure setup and dosimetry

#### 2.1.1 Generation and optimized delivery of 30.5 GHz CW waves *in vitro*

**Figure 2:**
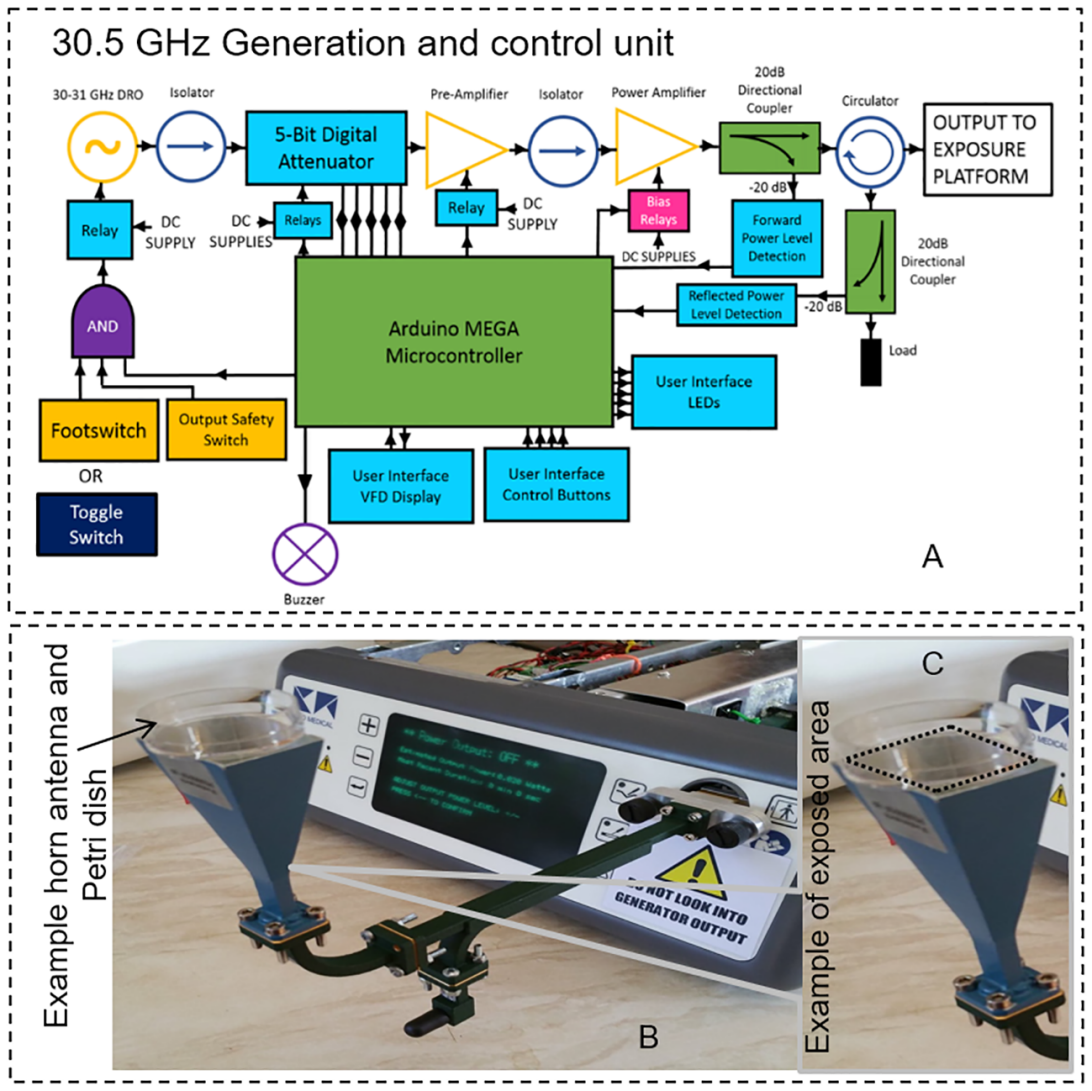
**(A)** Generation setup scheme of the system with power amplifier and power detectors for forward and reflected power **(B)** the final generator and control unit connected to the horn antenna using a standard waveguide at 30.5 GHz is shown. **(C)** An example of exposed area in a Petri dish is shown in the figure inset.

A MMW generator was expressly developed for the scheduled bio-experiments (Creo-Medical, UK). A maximum theoretical output power of 5 W (37 dBm) was initially hypothesized to compensate for power attenuation throughout the output transmission line and ensure enough energy at the exposure platform for a range of biological experiments. Individual components were chosen to meet or exceed the requirements of the design. The chosen oscillator can be mechanically tuned between 30 and 31 GHz with a power output of approximately 11.5 dBm. A gain stage is used to amplify the signal from the oscillator providing a maximum power of 37 dBm at the PA output. Detectors in the forward and reflected signal paths provide a way to monitor the forward and reflected power levels by producing a DC voltage that is proportional to the MMW power level. An attenuator was included in the final design to allow more user control over the output power. This component and others, such as the PA, require specific digital control for operation implemented by a microcontroller **Figure 2A**. This allows the user to control the MMW attenuation level, read the forward and reflected power levels on screen and set the duration of the power delivery, **Figure 2B**. The microcontroller responds to requests from the user via control buttons and outputs data to the display screen. Controlling and monitoring these parameters is essential for undertaking and replicating biological experiments. The generator is housed in its own enclosure and is powered from mains 85 – 250 V AC. The output connector is a “quick-release” waveguide (WG22) which connects to the exposure platform (a horn antenna) via flexible waveguide. The overall maximum power output is approximately 4.5 Watts (36.5 dBm). With this setup (**Figure 2C**) organoids were exposed in two modalities named: CW1 incident power of 0.1 W (RMS) for 20 minutes and CW2 incident power of 0.2 W (RMS) for 10 minutes. Sham groups were included in the experiments: sham samples received the same treatment as the exposed ones but no MMW power was active in this case.

#### 2.1.2 Numerical dosimetry: EM and thermal characterization

**Figure 3:**
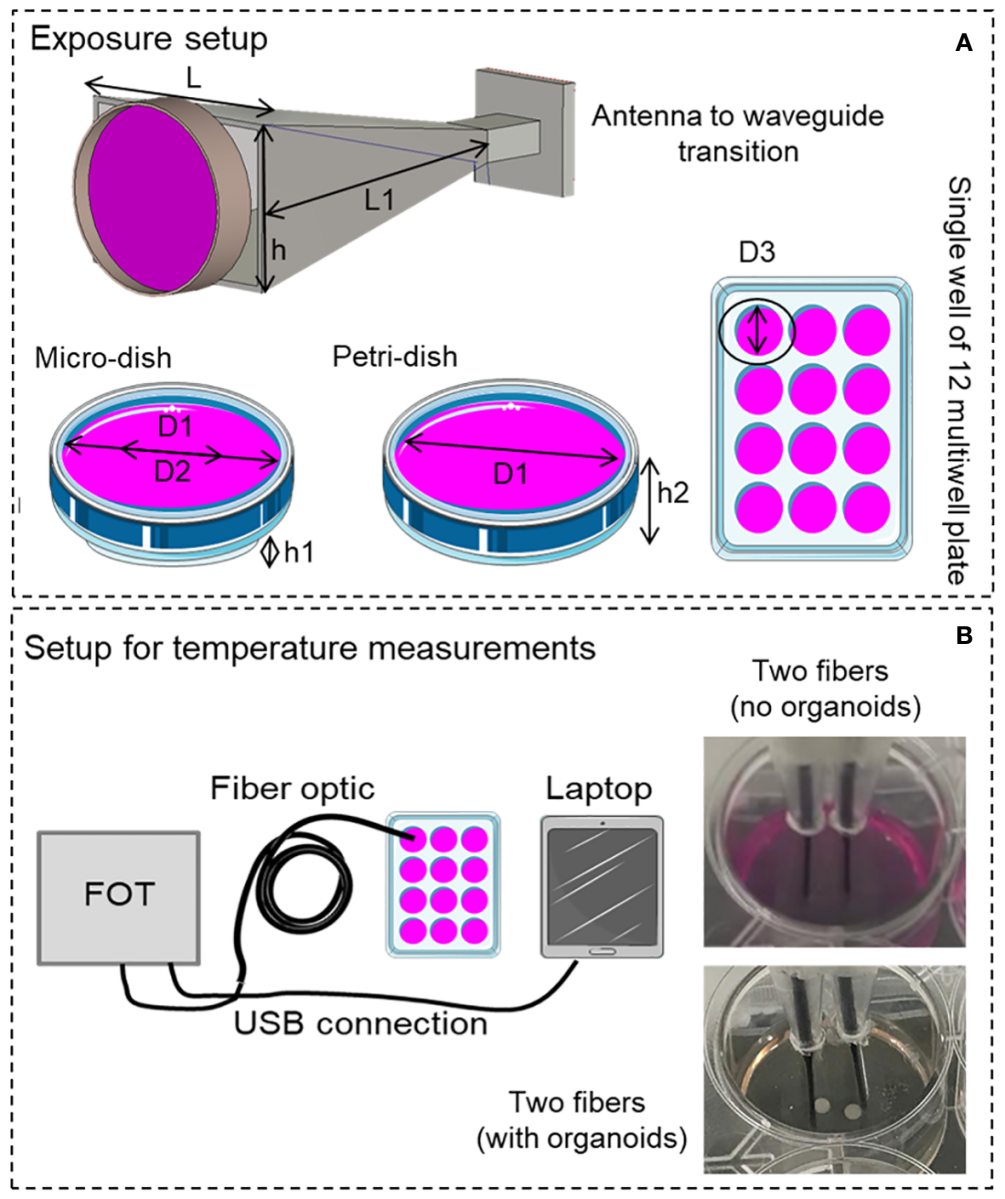
**(A)** Simulated horn antenna with dimensions indicated in **Table 1**, different biological containers were taken into account in simulations: micro-dish, Petri-dish and a single well of a 12 multiwell plate. **(B)** Setup for temperature measurements, measurement points using the two probe channels are observable in the figure inset in presence and absence of GBM organoids.

Dosimetric assessment was numerically performed by EM simulations using CST Microwave Studio (v.15). The horn antenna dimensions are reported in **Table 1**, together with dimensions of the different simulated sample holders, see also **Figure 3A**. The first step was to choose the best sample holder type (and dimensions) in terms of reflection coefficient (S_11_) and Specific Absorption Rate (SAR) inhomogeneity minimization (8, 21, 22).

**Table 1:** Horn antenna dimensions together with biological sample holde dimensions used in numerical simulations.

Letters as in Figure 3A	Dimension (mm)
H	27.8
h1	2
h2	7
L	36.8
L1	77.9
D1	32
D2	21
D3	22

Additional simulations were performed to optimize the culture medium volume and the best distance of the holder from the exposure antenna looking at the minimization of the SAR inhomogeneity. SAR inhomogeneity was computed as the ratio between the standard deviation of the SAR over the chosen averaging volume divided by the SAR mean value on the same volume, known as coefficient of variation (CV) (22–25).

Three biological containers were taken into account: a 35-mm-diameter Petri dish, a so called “microdish”: a 35-mm diameter Petri dish container having a smaller and thicker base section adapted for inverted microscopy, and a single well of a 12 multiwell plate mimicking the exposure of the single well into the plate.

The culture medium volume was optimized considering different liquid heights from 1 to 5 mm with steps of 1 mm (corresponding to different culture medium volumes). Finally, EM simulations placing the holder at different distances from the horn antenna were carried out (at 0, 10, 20, 30 40 and 50 mm from the antenna aperture).

Meniscus effect was also included into the model as reported in (26). Permittivity and tgδ of the different simulated materials at 30.5 GHz are reported in **Table 2**, the antenna metal was simulated as Perfect Electric Conductor.

**Table 2:** Electric parameters of simulated materials at 30.5 GHz.

Material (at 30.5 GHz)	ε	tgδ
Polycarbonate	2.9	0.01
Cell culture medium	27	1.27
Brain white matter	20	0.3

After evaluation of optimal exposure conditions in the absence of the GBM organoids, EM and coupled thermal simulations were performed to evaluate SAR in the organoids. Organoids were simulated as cylinders with a diameter of 2 mm and a height of 0.7 mm, two organoids spaced by 5 mm were placed in the holder center as in the actual exposure conditions. EM properties of the organoids considered as white matter are reported in **Table 2**. SAR levels in the organoids were evaluated slice by slice at steps of 100 µm. Coupled thermal simulations (Multiphysic transient thermal solver) allowed the evaluation of global time trend of temperature distribution into the sample holder in the presence of the organoids, thermal parameters for the analysis are reported in **Table 3**. Total mesh cells were 169×10^6^ with a minimum dimension of 20 µm, simulations run on Work Station Intel ^®^ Xeon ^®^ with two CPUs and 128 GB of RAM, lasting around 3 hours each.

**Table 3:** Thermal parameters of simulated materials.

Material (at 30.5 GHz)	Thermal cond. (W/k/m)	Density (kg/m^3^)	Specific heath (J/K/kg)
Polycarbonate	0.19	1200	1200
Cell culture medium	0.6	1000	4184
Brain white matter	0.5	1030	3600

#### 2.1.3 Experimental dosimetry

**Figure 4:**
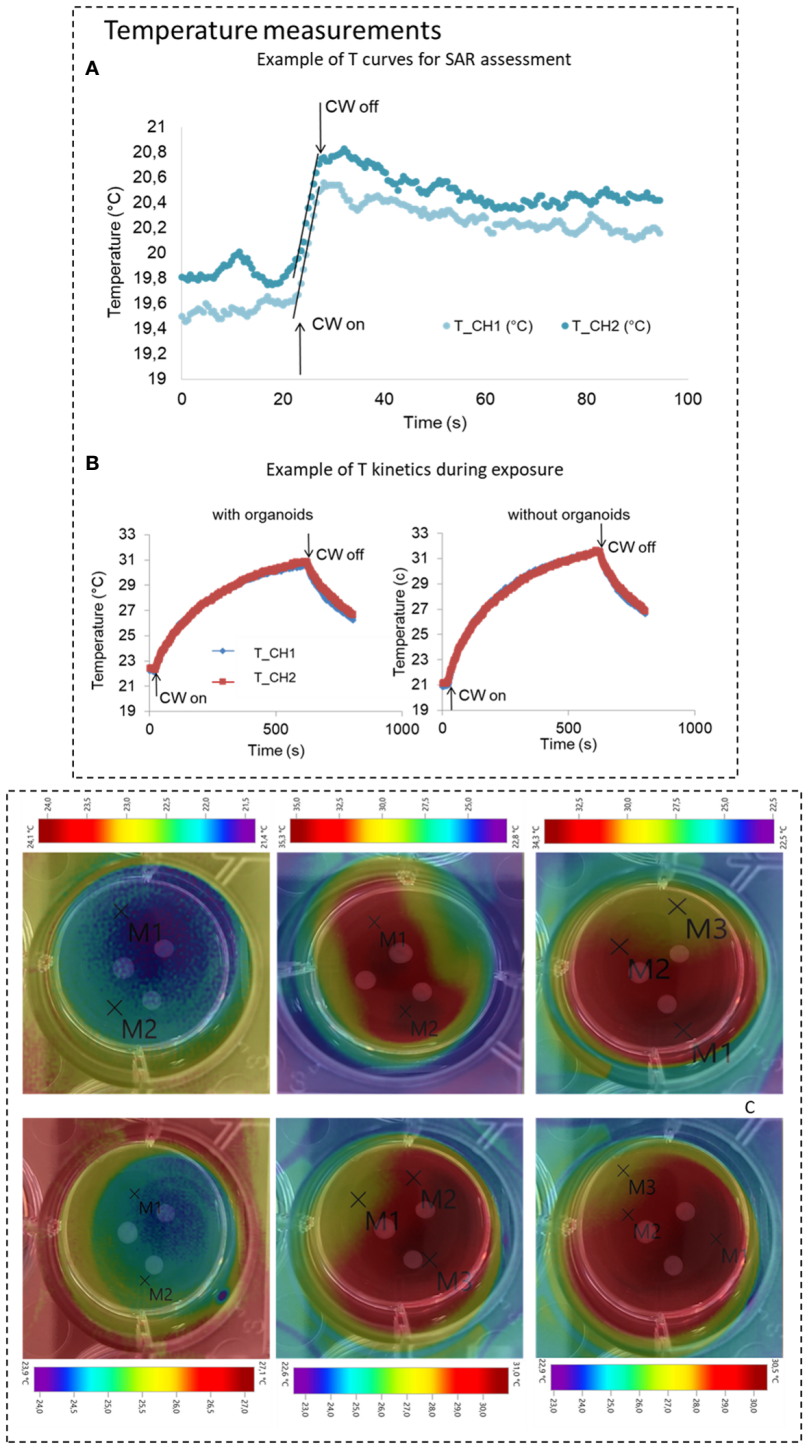
**(A)** Temperature measurements for SAR assessment, top panel, example for and Winc of 1.24 W (RMS), the linear regression for SAR calculation is highlighted. **(B)** Example of temperature kinetics during experiments in presence or absence of organoids are also reported in the bottom panel (Winc of 0.2 W RMS). **(C)** Temperature measurements from thermo-camera acquisitions at three different instants at the start during and at the end of the exposure time for both electromagnetic protocols. Images present superposed to the thermal maps the photo of the single well where the exposed GBM organoids are visible (for these measurements three organoids were taken into account). In the images temperature markers are also visible.

SAR measurements using non-perturbing thermometric methods (8, 23) were carried out to experimentally assess SAR in order to validate our numerical model in the absence of the organoids into the culture well. Temperature measurements were performed using a fiber optic probe (Lumasense, US, uncertainty ±0.1°C) with two sensors per well located 5 mm apart from each other, see **Figure 3B**. Temperature measurements, accomplished using the cell holder and liquid volume that minimize SAR inhomogeneity (detailed in Results section 3.1), were performed using the 30.5 GHz generator (section 2.1.1) at three different powers (0.87, 1.02, 1.24 W RMS) and 24 independent measurements were achieved over the two channels for each power level. The culture medium was jellified adding 4% of agar to avoid liquid evaporation and biases due to convection effects during temperature measurements. SAR was assessed following the well know procedure as fully detailed in (23, 24). Briefly, at the thermal equilibrium EM power was switched on for 2 s and the temperature increase was measured over this short time (to avoid convective and radiated phenomena). A robust average algorithm was used to mediate all the acquired data for each delivered power and to interpolate temperature data during the 2 s of exposure using a linear regression method (23, 24, 26), see **Figure 4A** (top panel) for the measured temperature increment. The protocol included two further dwell times before and after the millimeter wave exposure to guarantee temperature stability at the beginning of the exposure, and to verify temperature decay once the MMW power was turned off. A Labview code enabled a PC with and Serial interface to automatically acquire temperature, **Figure 3B**. Power monitoring during exposure was performed using the generator monitoring option as described in section 2.1.1. Two 3-mm-diameter holes were made on the top of the multiwell container to enable precise probe insertion. In **Figure 3B** details of the apparatus for temperature measurements are reported.

#### 2.1.4 Thermal assessment of the samples

To determine the thermal behaviors of the target in actual biological experiments a set of temperature measurements were carried out in presence and absences of the organoids in the well. Temperature measurements were performed using the same measurement setup above described (**Figure 3B**) and EM powers of 0.1 and 0.2 W RMS. A different protocol was used, which includes 20 and 10 minutes of 30.5 GHz exposure for the two powers respectively. To assess the global distribution of the temperature into the well, not only at the two measurement points as using the fiberoptic probes, temperature measurements were also performed using a thermo-camera (Testo 875–2i). The images were acquired at the exposure starting (T=0), during the exposure (T=10 min for CW1 protocol and T=5 min for CW2 protocol), and at the end of the exposure for both protocols. Temperature increments for the two protocols were extracted from three independent experiments (**Figures 4B, C**).

### 2.2 Cellular biology procedures

#### 2.2.1 Organoids preparation

Three different primary, patient’s derived GBM cultures (HuTuP13, HuTuP61 and HuTuP176), already established and characterized within previous studies, were cultured as previously described (27, 28). GBM patient-derived organoids were generated by resuspending 10^5^ cells in 10µl of Matrigel (BD Biosciences, Italy) and plated onto sterile dimpled parafilm mold for the generation of Matrigel droplets (29). After Matrigel polymerization, droplets were transferred to cell culture dish in DMEM-F12 growth medium containing the serum analogue BIT9500 (Voden, Stem Cells Technology, Italy) EGF and bFGF growth factors (Thermo Fisher Scientific, Italy) in an atmosphere of 2% oxygen, 5% carbon dioxide in a Don Whitley Scientific H35 hypoxic cabinet, to ensure a proper cell expansion in hypoxic conditions (30). Standard GBM culture protocol requires weekly half-medium changes to replace nutrients and growth factors and ensure optimal cell fitness. GBM organoids remained in culture for 21 days before CW exposure. Where indicated, sham exposed and CW exposed organoids were treated for 120 hours with 500µM TMZ or equivalent (v/v) vehicle (DMSO).

#### 2.2.2 Transcriptional profiling and real time quantitative PCR

Gene expression profiling of Sham- and CW-exposed GBM organoids was performed 24 hours post exposure through Clariom S Affymetrix Gene Chips (Affymetrix, Thermo Fisher Scientific, Waltam, MA, USA). In particular, *in vitro* transcription, hybridization and biotin labeling of RNA were performed according to Gene Chip™ WT Kit protocol and Clariom™ S human gene platform. Microarray data (CEL files) were generated using default Affymetrix microarray analysis parameters (Command Console Suite Software by Affymetrix, Italy) and then normalized by Repeated Measure Analysis (RMA) through the TAC software (Affymetrix). Differentially expressed genes in Sham and 30.5 GHz exposed (CW1 and CW2) samples (N = 4 for each group) were identified using Limma (FDR<0.05 and fold change ≥2) (31). Expression data were deposited into the Gene Expression Omnibus (GEO) database under Series Accession Number GSE239486 and are accessible without restrictions. In order to identify the potential pathways and intracellular signaling affected by 30.5 GHz CW exposures (CW1 and CW2), we performed Gene Set Enrichment Analysis in the C2cp gene set collection and then generated an Enrichment Map of the significantly enriched C2cp GSEA terms (FDR ≤ 0.01) by using the Enrichment Map application in Cytoscape 3.10.0.

RQ-PCR of Sham- and 30.5 GHz exposed (CW1 and CW2) GBM organoids was performed 24 hrs post exposure. In particular, 500 ng of total RNA were reverse-transcribed using the Reliance Select cDNA Synthesis Kit (Bio-Rad, Hercules, CA) and the quantitative RT-PCR reactions were run in triplicate using Platinum SYBR Green Q-PCR Super Mix (Thermo Fisher Scientific, Waltham, MA). Fluorescent emission was recorded in real-time (QuantStudio™ 5 Real-Time PCR, Applied Biosystems, Foster City, CA). The specificity of primers was confirmed for every PCR run by dissociation curve analysis. Primers used are listed in **Supplementary Table 1** and specificity confirmed by Human BLAT Search (http://genome.ucsc.edu). Relative RNA quantities were normalized to GUSB expression according to the ΔΔCt Method.

#### 2.2.3 Histology and immunofluorescence

Sham- and CW-exposed GBM organoids, at pre-determined time points (24, 72 or 120 hrs), were fixed in cold 4% formaldehyde for 30 minutes, rinsed and then cryoprotected in sucrose 30% for 20 hrs. GBM organoids were then embedded in OCT and stored at -80°C until being cryosectioned for histology and immunofluorescence analyses. Hematoxylin and eosin staining was performed according to standard procedures and images were captured with a Nikon SMZ100 microscope (Nikon, Melville, NY).

Immunofluorescence was performed through standard procedures. Briefly, slides were re-hydrated in PBS, blocked in a 5% BSA (Sigma-Aldrich, St. Louis, MO), 1% goat serum (Vector Laboratories, Newark, CA) PBS solution, and then stained with primary antibodies: Ki67 (1:100); GFAP (1:500; both from Agilent Technologies, Santa Clara, CA); Cleaved-Caspase 3 (1:200; Cell Signaling Technology, Danvers, MA). Appropriate Alexa-dye conjugated secondary antibodies were used (1:200, Life Technologies, Carlsbad, CA). Finally, samples have been counterstained with DAPI (1 μg/ml, Sigma-Aldrich, Saint Louis, MO). Images were collected with a Zeiss Axio Imager M1 epifluorescence microscope (Zeiss, Oberkochen, Germany). Images were analysed and cellular nuclei were counted by the Analyze plugin of ImageJ (https://imagej.nih.gov).

#### 2.2.4 Statistical analysis

Data in graphs were analyzed using statistical tools provided within GraphPad Prism 8.0.1 software (GraphPad, La Jolla, CA). Bar graphs display data arranged as mean ± standard error of the mean (S.E.M.). One-way ANOVA with Tukey’s multiple comparison test was used for comparing data from three or more experimental groups. In addition, we used t-test for comparing two groups. Asterisks indicate a statistically significant difference with sham cells (over bars) or selected experimental groups (over brackets, when present). In particular, * p ≤ 0.05, ** p ≤ 0.01, *** p ≤ 0.001, **** p ≤ 0.0001.

## 3 Results

### 3.1 Validation of numerical simulations, 30.5 GHz CW exposure optimization and thermal assessment

First, numerical simulation performed were validated by comparing simulated and measured SAR values. The achieved SAR values from measurements (921 W/kg for 0.5 W of incident power RMS) and simulations (1010 W/kg for 0.5 W of incident power RMS) showed a variation of 10% which is in the range of the measurement standard deviation (see **Table 4**, SAR without organoids), hence validating the performed simulations in the absence of the organoids in the single well.

**Table 4:** Measured and simulated SAR values (average volume 1 mm^3^).

Power (W RMS)	SAR measured (average volume 1 mm^3^)	SAR simulated (average volume 1 mm^3^)
0.1 (without organoids)	63	69
0.1 (with organoids)	51.0	58
0.2 (without organoids)	118.3	153
0.2 (with organoids)	113.1	125.7

**Figure 5:**
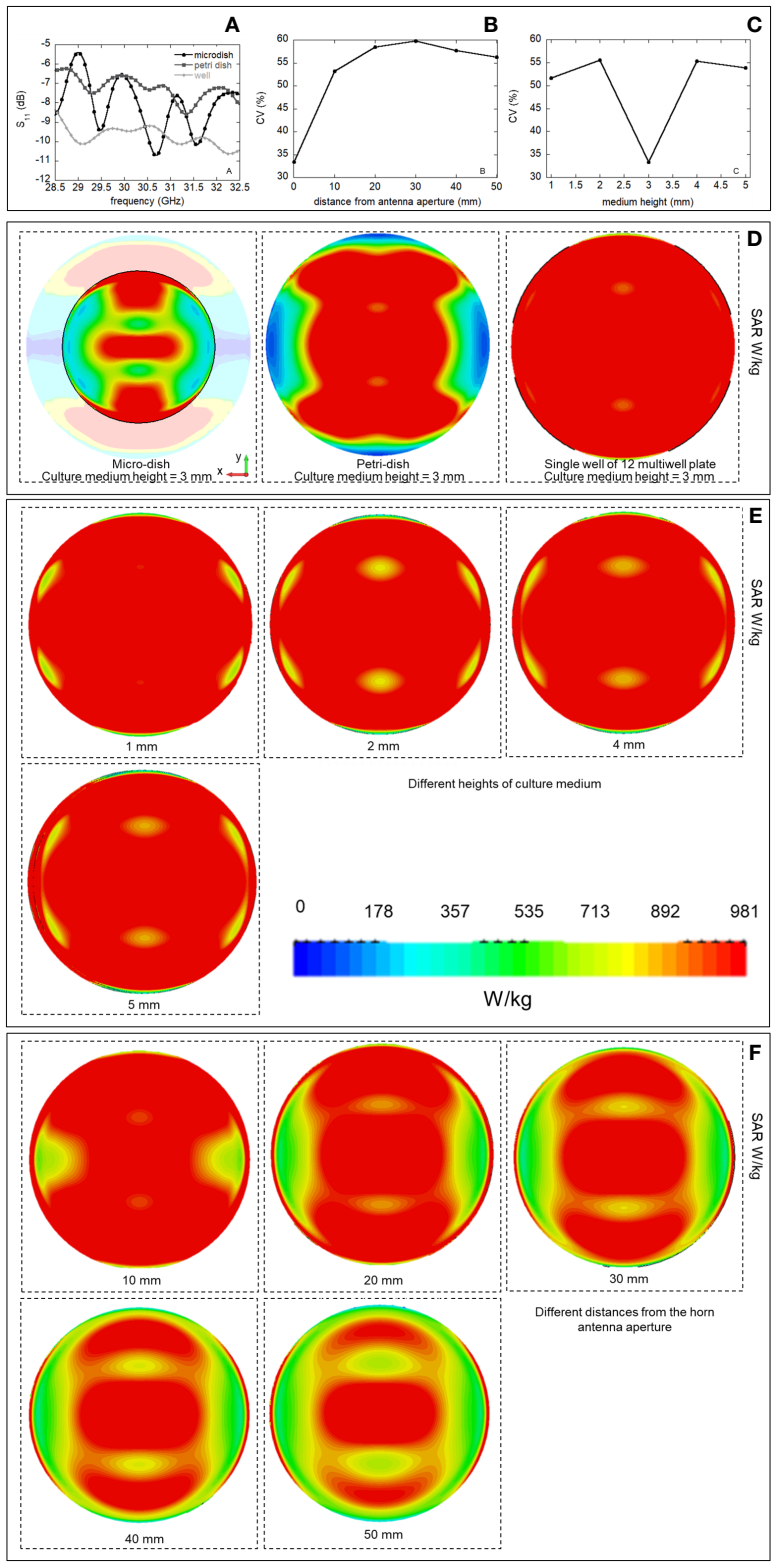
Reflection coefficients (S_11_, dB) for the different biological containers placed directly on the plane of the antenna aperture **(A)**, CV in % (ratio between the SAR standard deviation and the mean value averaged over an height of 100 µm from the bottom of the well) as a function of the height of the culture medium into the well, minimization of the CV is achieved using a medium height of 3mm corresponding to a medium volume of 700 µl **(B)**. CV in % as a function of the distance of the well from the antenna aperture, minimization of the CV is achieved for the well placed on the antenna aperture **(C)**. SAR spatial distribution for 0.5 W of incident power (RMS) on the horn antenna aperture for the different biological containers, placed directly on the plane of the antenna aperture (medium height of 3 mm), **(D)**, for different heights of the cell culturing medium in the single well **(E)**, for different distances of the single well of 12 multiwell plate from the horn antenna **(F)**.

Once the numerical model was experimentally validated it was used to drive optimization of the exposure. Among the different exposure modalities investigated, the best one was achieved for the horn antenna exciting the single well, as shown in **Figure 5A**, reporting the S_11_ parameters for the different simulated containers. Comparable S_11_ values (around -9.5 dB) were achieved for the microdish and the well at 30.5 GHz, demonstrating good matching of these two specific structures. In our case, the well was chosen for its easy handling and lower cost in biological experiments. SAR inhomogeneity expressed as CV in percent (%) was also evaluated up to 100 µm of height from the bottom of the containers, as shown in **Table 5**. The lowest CV value was obtained for the well, confirming this as the optimal irradiation condition (**Table 5**). Spatial SAR distributions at the bottom (XY plane) of the containers at 30.5 GHz are also shown in **Figure 5D**, where the most homogeneous SAR distribution for the well with respect to the other containers was achieved. To run these simulations the medium height was kept to 3 mm.

**Table 5:** CV values for different biological container.

CV containers	
Type	Value (%)
Micro-dish	52.3
Petri dish	52.9
Well	32.8

EM simulations were then used to assess the best liquid volume in terms of its height into the well, as presented in **Figure 5B** for CV coefficient in %. 3 mm of liquid height corresponding to a volume of 700 µL was found to minimize the CV, guarantying maximal SAR homogeneity within the cell culture medium. SAR distributions at the well bottom (on XY plane) are also reported at 30.5 GHz in the different simulated conditions, **Figure 5E**.

Finally, EM simulations defined the best distance of the horn antenna with respect to the well as observable in **Figure 5C**, where it is shown that the CV in % is minimized for the well placed in direct contact with the horn antenna. SAR distributions at the well bottom (on XY plane) are also reported at 30.5 GHz, in the different simulated conditions in **Figure 5F**.

### 3.2 Numerical and experimental dosimetry characterization and validation

**Figure 6:**
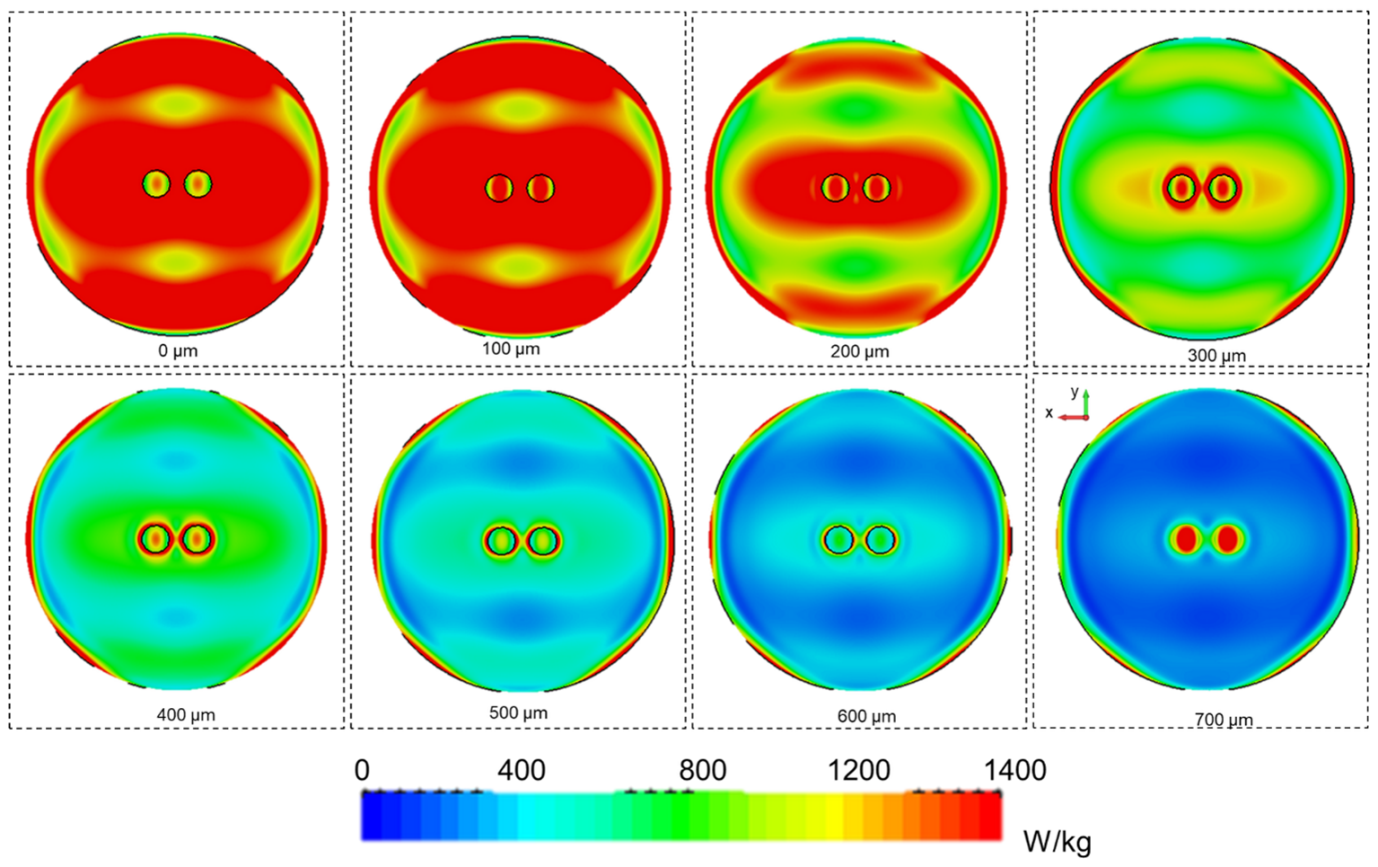
SAR spatial distributions for 0.5 W of incident power (RMS) on the horn antenna aperture for the well containing 2 organoids. SAR distributions on the XY planes are reported at different heights from the bottom of the well (0 µm) up to 700 µm at steps of 100 µm.

After the validation of the numerical model in the absence of organoids, the SAR inhomogeneity over the entire organoid volume was computed. In the first 100 µm of the organoids, the CV was 27% (SAR of 340 W/kg for an incident power of 0.1W RMS) whereas a CV value of nearly 70% (SAR of 60 W/kg for an incident power of 0.1W RMS) was demonstrated at the maximum organoid height. In **Table 6**, results for the further analyzed volumes are shown presenting SAR inhomogeneity in terms of CV values between the above mentioned range depending, as expected, on the distance of the considered organoid layer from the bottom of the container. In **Table 4**, the measured and simulated SAR values for the two exposure powers respectively (0.1 and 0.2 W RMS) with organoids are presented for comparison. Good agreement was found between simulated and measured data. For a correct comparison, SAR values in simulations were averaged across a volume comparable to the one used for temperature measurements, i.e., a 1-mm side cube. In **Figure 6**, SAR spatial distributions for the different organoid layers were reported inside the well looking at the XY plane for an incident power of 0.5W RMS. In the same figure, the SAR distribution within the organoids shows distinct hotspots, with the highest absorbance at the center of the organoids, gradually decreasing towards the edges. This is due to dielectric discontinuities between the organoid and the medium, leading to differential absorption of MMW energy. A similar disruption in the uniformity of SAR is observed along the edges of the well. Similarly to what previous observed along the organoid edges, this is again due to reflections at the interface between the well and the surrounding medium. Furthermore, the SAR distribution pattern within the well appears more similar to that of the medium at a certain height (5 mm, see **Figure 5E**) or at a certain distance (30 mm, see **Figure 5F**) from the horn antenna. This suggests that the presence of the organoids within the well is altering the propagation of electromagnetic fields, causing the SAR distribution to resemble that of a different medium heights or antenna distances. Overall, these observations highlight the complex interactions between organoids and electromagnetic fields, hence demonstrating the relevance of performing a careful dosimetry also in the presence of the biological targets.

**Table 6:** CV values for different organoid layers at a step of 100 µm.

Organoid layer thickness (µm)	CV in organoids (%)
100	27.6
200	30.3
300	34.5
400	40.9
500	48.8
600	58.0
700	69.5

The temperature was then monitored, with the optical sensor, for the entire duration of the two exposure protocols CW1 and CW2. These measurements were performed in the presence and absence of the two organoids. Examples of acquisitions are reported in **Figure 4B** (central panel) for CW2 exposure: measured temperature increase was equal to 7.2± °C. For the CW1 exposure, there was an average increase of 4.5± °C. These variations were also confirmed by coupled EM and thermal simulations that predicted simulated thermal variations of 4.4 and 8.8°C in the same measurement points for CW1 and CW2 respectively (see **Figure 3B**). Measurements from thermo-camera, presented in **Figure 4C**, show a homogeneous distribution of the temperature, no hot spots are visible in proximity of the GBM organoids with temperature increases of 9.5 and 3.8°C for CW2 and CW1, respectively, in line with the optical fiber measurements and data from thermal simulations. These data suggest that the observed temperature increase is mostly related to the delivered power rather than the exposure time or the delivered energy.

### 3.3 Transcriptional profiling: sham versus CW exposed organoids

**Figure 7:**
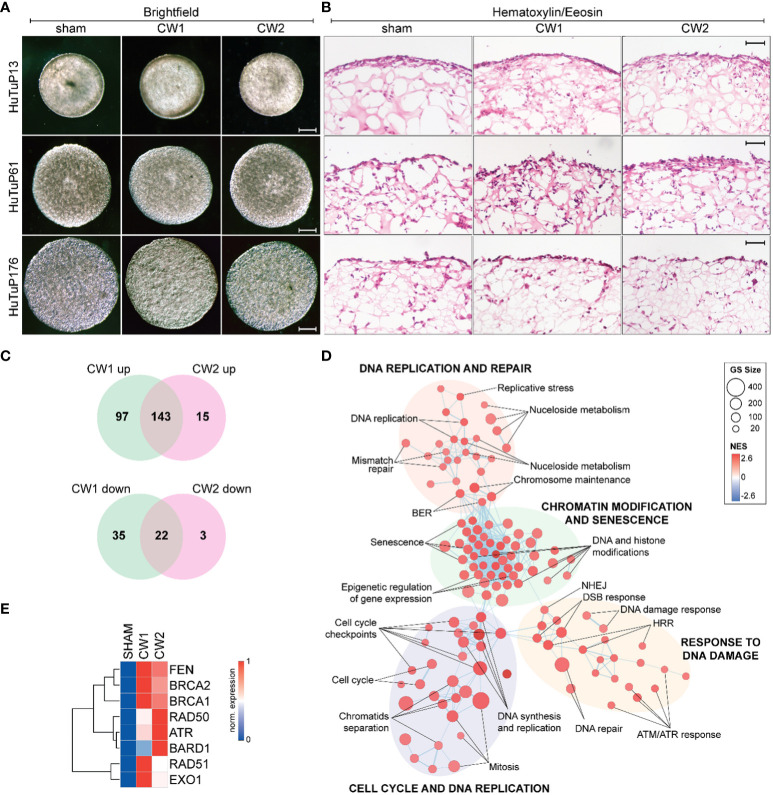
**(A, B)** Representative bright field **(A)** and histological (hematoxylin and eosin staining) images of GBM organoids, generated with HuTuP13, 61, and 176 primary cells, after 24 hrs from being exposed to sham, CW1, or CW2 protocols. **(B)** Original magnification: 3.2x; bar: 500µm. **(C)** Venn diagrams summarizing the number of common differentially expressed genes (up-regulated and down-regulated in upper and bottom panel, respectively) between CW1/CW2 and control sham treated GBM organoids. **(D)** Enrichment map displaying the significantly (FDR q value<0.01) enriched pathways (C2cp MSigDB) in CW1/CW2-treated GBM organoids relative to matched controls. **(E)** Heatmap summarizing the expression of selected DNA damage response genes in sham and CW-treated GBM organoids (24 hrs) by RQ-PCR. GS: Gene set; NES: Normalized Enrichment Score; HRR: Homologous Recombination Repair; DSB: Double Strand Break; NHEJ: Non-Homologous End Joining; BER: Base Excision Repair; Norm: normalized.

**Figure 8:**
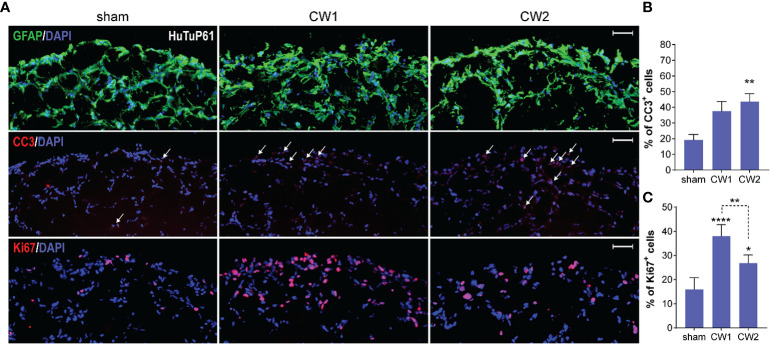
**(A)** Immunofluorescence analysis displaying representative control and CW-treated (72hrs) GBM organoids (HuTuP61) stained for GFAP (green, upper panels), Cleaved Caspase 3 (CC3; red, middle panels), and Ki67 (red, lower panels). Original magnification: 10x; bar: 50µm. **(B, C)** Relative quantifications of CC3+ **(B)** and Ki67+ **(C)** cells in HuTuP13, 61, and 176 GBM organoids treated as indicated (72hrs). *p<0.05, **p<0.01, ****p<0.0001 by one-way Anova with Tukey’s multiple comparisons test. An asterisk over a column indicates a significant difference relative to the sham group.

Once the technical experimental conditions were carefully set up, we went further in exploring and characterizing the biological effects of CW1 and CW2 signals. GBM cells from three different primary patient-derived specimens were cultured under hypoxic conditions (2% O_2_) in a serum-free medium enriched for neural specific growth factors (see section 2.2.1). These restricted culturing conditions resemble the more physiological brain tumor microenvironment in which GBM cells grow, with a specific focus in the maintenance of their stem cell fraction. GBM organoids were prepared according to the previously described procedures (see section 2.2.1) and then grown in standard culturing conditions (21% O_2_) for additional 21 days. Within this set up we exposed GBM organoids to CW1 and CW2. As depicted in **Figure 7A**, we could not detect any structural and morphological difference in exposed organoids neither at 24 hrs post exposure nor after 72 hrs (**Figure 7A** and **Supplementary Figure S1A**), when compared to their relative controls (sham). Moreover, histological analysis performed at 24 and 72 hrs post-exposure revealed that both CW1 and CW2 did not alter the structural tissue morphology of the organoids. No evidence for necrotic areas was observed within the inner portions of the organoid structure being the majority of damaged cells located in the outer layer (**Figure 7B** and **Supplementary Figure S1B**). In order to evaluate possible changes in activation status of any intracellular pathway we subjected sham and CW1/CW2 organoids to whole transcriptome analysis 24 hrs after exposure. Strikingly, CW-exposed organoids displayed a clear-cut difference in term of transcriptional features relative to sham exposed organoids. We were able to identify 257 differentially expressed genes (DEGs) between sham and CW1 exposed organoids, and 183 DEGs between sham and CW2 exposed organoids. Since the delivered energy was equal in the two different treatments, we aimed to identify the common perturbed genes; by intersecting the two different gene lists we retrieved 143 upregulated genes and 22 downregulated genes commonly affected by 30.5 GHz CW exposure (**Figure 7C**). Pathway enrichment analysis through GSEA identified CW-exposed organoids as characterized by an increased expression of genes related to: i) cell cycle and DNA replication; ii) response to DNA damage; iii) chromatin modification and senescence; iv) DNA repair (**Figure 7D**). More specifically, the majority of the up-regulated DEGs were related to the regulation of chromatin conformation and replication machinery. Moreover, we found a clear-cut up-regulation of the main DNA repair pathways, including DNA repair, double strand break response (DSB), homologous recombination repair (HRR) of DNA, and senescence, thus suggesting a possible DNA damaging effect exerted by CW exposure (**Figure 8D**). The few down-regulated genes did not significantly enrich for any cellular process. To validate the transcriptional changes associated with the CW exposure, particularly the DNA damage response, we selected eight DNA repair associated genes and evaluated their expression in response to CW1 or CW2 stimulation by RQ-PCR. We report a strong and significant upregulation of the DNA damage and repair machinery genes in response to both CW1 and CW2 exposure protocols (**Figure 8E**), confirming the potential DNA damaging effect exerted by the exposure of 30GHz CWs. Further, we evaluated if the above described transcriptional perturbation was accompanied by phenotypical effects in terms of cellular differentiation, induction of apoptosis, and proliferation. Since GBM organoids are able to recapitulate the tissue 3D structure of a tumour mass (20), we histologically evaluated the expression of specific GBM markers by immunofluorescence. At 24 hrs we could not detect any difference in the astrocytic differentiation marker Glial Fibrillary Acidic Protein (GFAP) between CW exposed and sham organoids post exposure in any of the primary GBM cells analyzed (**Supplementary Figure 2A**). Moreover, we could not detect any perturbation of cell proliferation through the staining with the well-known proliferation maker Ki67. Finally, the apoptotic mediator caspase 3 (CC3) displayed a trend toward its increased activation by both CW1 and CW2, although without displaying a statistically significant variation (**Supplementary Figures 2A-C**).

### 3.4 Long term effects of organoid exposure

Starting from the finding that both CW1 and CW2 exposure perturbed the transcriptional profile of GBM organoids at 24 hrs, we investigated the medium-term (72 hrs) effects of CW exposure with respect to cellular differentiation, induction of apoptosis, and proliferation. At this time point, we still observed no differences in terms of GFAP expression and distribution between CW exposed and sham organoids (**Figure 8A** and **Supplementary Figure S3**). However, by analysing the activation of the pro-apoptotic marker CC3, we found a significant increase of apoptosis in all the three organoid models exposed to CW2; CW1 exerted a clear, although less significant, increase of CC3 levels (**Figures 8A, B** and **Supplementary Figure S3**). In parallel, we also identified a significant increase in the amount of Ki67^+^ cells, for both CW1 and CW2 exposures compared to sham samples, in accordance with gene expression data (**Figures 8A, B**).

**Figure 9:**
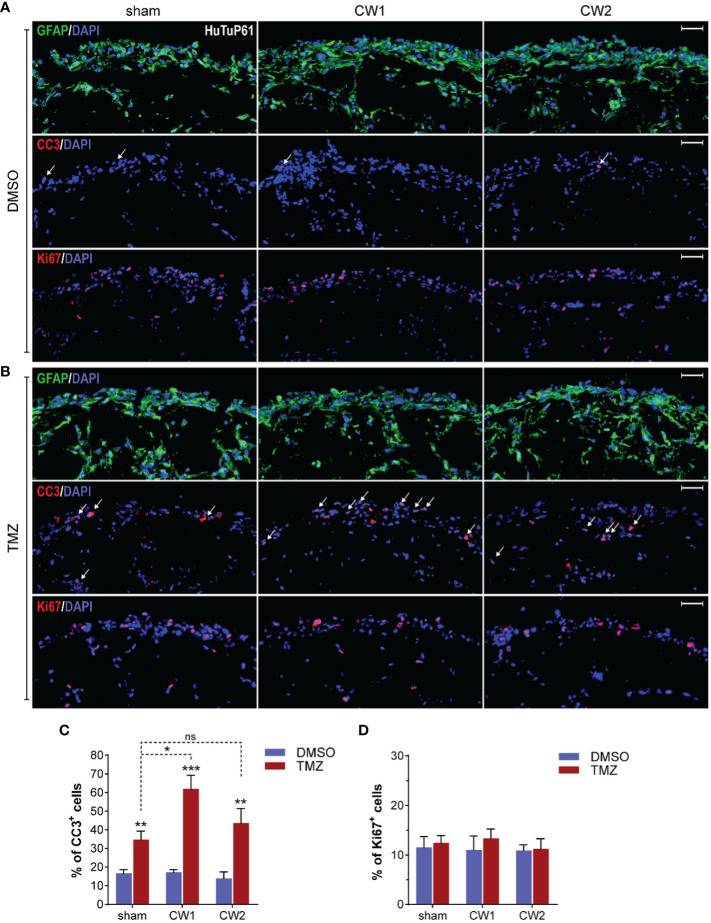
**(A, B)** Immunofluorescence analysis displaying HuTuP61 GBM organoids treated for 120hrs with DMSO **(A)** or TMZ **(B)** both added to the culturing medium 24hrs from sham or CW1/CW2 protocol exposure. Organoids have been stained for GFAP (green, upper panels), Cleaved Caspase 3 (CC3; red, middle panels), and Ki67 (red, lower panels). Original magnification: 10x; bar: 50µm. **(C, D)** Relative quantifications of CC3+ **(C)** and Ki67+ **(D)** cells in HuTuP13, 61, and 176 GBM organoids treated as in **(A)**. *p<0.05, **p<0.01, ***p<0.001 by one-way Anova with Tukey’s multiple comparisons test. An asterisk over a column indicates a significant difference relative to matched DMSO-treated groups. ns: not significant.

Data collected depict a quite paradoxical pictures in which CW exposure induces a concomitant early increase of both cell death and proliferation at 72 hrs. Interestingly, since the observed CW-dependent increase in cellular proliferation could be exploited as a major sensitizing factor to alkylating agents, such as TMZ, in order to evaluate if any increased biological and therapeutic effect would occur upon this combined treatment, we exposed GBM organoids to CW1 and CW2 schedules. The day after, we treated them with 500µM TMZ (or vehicle, DMSO) for additional 5 days. Immunofluorescence analysis revealed that CW exposure significantly increased the pro-apoptotic effect of TMZ in GBM cells, with a stronger action exerted by CW1 (**Figures 9A-D**), consistent with a more potent induction of cell proliferation at earlier time points (**Figure 7C**). Moreover, we could not observe any CW-induced pro-apoptotic/proliferative effect in DMSO treated organoids (**Figures 9A-C**), this suggests that the previously reported increase of CC3^+^ and Ki67^+^ cells at 72 hrs (**Figures 7B, C**) could be dependent only on an early response of GBM cells to the CW stimulation, which then extinguishes if cells are not re-exposed. Accordingly, CW signals seem to open a restricted interventional window in which to treat cells (i.e. with TMZ) in order to exploit CW signal properties for potential therapeutic purposes. Finally, consistent with previous data, the CW/TMZ combination did not alter the expression of GFAP (**Figures 9A, B**).

## 4 Discussion

In this study, we investigated the potential effects of a 30.5 GHz CW signal for therapeutic purposes in GBM. The frequency was selected based on technologic opportunity, relative to previous work, and in the context of growing interest for MMW applications. While different frequencies within and beyond the Ka-band are legitimate alternatives, the approach in this work may lead to interesting findings in other regions of the MMW spectrum. Our dosimetric observations highlight the complex interactions between organoids and electromagnetic fields, hence demonstrating the relevance of performing such a rigorous analysis prior to experiments in order to optimize the choice of different parameters involved (e.g. type of biological container, culture medium, volume, distance from the feeding antenna).

Specifically, we focused our efforts on an extremely aggressive brain tumor GBM, which remains an incurable disease due its intrinsic resistance to therapies (32). Recent reports point at organoids as reliable compromises between *in vitro* and *in vivo* experiments as they represent a more complex model if compared to cell monolayers without the inconvenience of costs and ethical issues associated with animal models (33). It has been demonstrated that 3D organoids generated starting from human GBM patients may be considered as relevant models of the original tumor mass from which they have been derived, thus reproducing the most important features characterizing the disease such as the high cell heterogeneity and proliferation, together with a proper tumor microenvironment that can influence the cell phenotype and their response to external stimuli (20).

Our results demonstrate that CW exposed GBM organoids activate the DNA damage response pathway and the cellular machineries related to chromatin remodeling, senescence and DNA repair.

Both CW1 and CW2 exposure led to an increase of cell proliferation after 72hrs from exposure together with a slight, although significant increase in the apoptotic rate. This last phenomenon is more marked in cells exposed to the CW2 protocol as possibly dependent on the higher temperature increase contributing to cell killing. Supporting this hypothesis, Orlacchio et al. (8) detected the initiation of a heat shock response through the activation of Caspase-3 and phosphorylation of HSP2A as a function of the sample temperature increase in melanoma cells exposed to pulses at 60 GHz. However, as reported in **Figure 4**, we kept the working temperature within a hypo-thermal range, since the organoids temperature never exceed 32°C.

Apoptosis and senescence activation were also detected in another study on MMWs applied in the treatment of lung cancers using H1299 cells. In this study however, different frequencies and a much lower signal amplitude were used (34). In cells exposed to the CW1 protocol, instead, there was an evident statistically significant increase of cell proliferation. This highly proliferating GBM cells are the ones targeted by TMZ inducing a stronger toxicity to cell DNA under replication (35)].

It seems necessary to refine this combination (30.5 GHz exposure with CW1 protocol and TMZ) to maintain a precise window both for the administration of TMZ after the exposure and for the delivered EM protocol in terms of signal amplitude and duration enabling the maximization of tumor cell proliferation.

In this regard, further studies are needed to clarify the potential synergistic effects between EM radiation exposure and TMZ doses in order to optimize chemotherapeutic dosage and temporal windows for treatments. Moreover, future studies could also involve the use of different chemotherapeutic drugs with different mechanisms of action, rather than the direct effect on proliferating cells to maximally increase the effect between chemotherapeutics and EM protocols and ultimately target tumor cells under non-proliferative conditions.

Other important biological players such as the ability of cells to migrate and invade the healthy tissues may also represent interesting fields of future investigations for the combined effects of 30.5 GHz CW exposure and chemotherapeutic drugs as other fundamental features in GMB treatment.

Other points to be analyzed could be also the activation of innate immune response occurring immediately after the activation of DNA damage pathways and the inflammation role after the MMWs exposure. In this context, a recent study using MMWs at a different frequency, but at a comparable power density, showed deregulation of the intracellular metabolomics profile of keratinocyte HaCat cells, independently from the temperature increase (36).

Our study is unique with respect to the exposure protocol and the GBM model used and no characterizations of the combination of MMWs and chemotherapeutic drugs are available so far. MMWs are suitable for their use in brain cancers due to their high spatial focusing and the possible application by endoscopic tools, even through robotic surgery approaches.

## 5 Conclusions

In summary, we comprehensively characterized an exposure setup for 30.5 GHz CW suitable for experiments on 3D GBM organoids, in terms of numerical/experimental dosimetry and thermal regimen.

From our characterization, we demonstrated the possibility to use this exposure modality (especially CW1 protocol) as a therapeutic adjuvant in a future possible treatment for GBM. Our approach impacts the functioning and behavior of GBM cells by inducing a statistically significant increase of proliferation, as confirmed by both transcriptional and functional analyses. This result leads to the potentiation of a subsequent TMZ administration.

These results might open the way to future and innovative combination therapies for GBM. This approach might be useful for targeting GBM cancer stem cells and improving their targeting, which remain extremely difficult so far.

## Data availability statement

The datasets presented in this study can be found in online repositories. The names of the repository/repositories and accession number(s) can be found below: https://www.ncbi.nlm.nih.gov/geo/, GSE239486.

## Ethics statement

The studies involving humans were approved by Department of Women’s and Children’s Health (SDB), University of Padova. The studies were conducted in accordance with the local legislation and institutional requirements. The participants provided their written informed consent to participate in this study.

## Author contributions

ER: Conceptualization, Data curation, Investigation, Methodology, Visualization, Writing – original draft. LP: Conceptualization, Data curation, Methodology, Software, Visualization, Writing – original draft. NK: Data curation, Investigation, Software, Validation, Visualization, Writing – review & editing. GH: Data curation, Formal analysis, Software, Validation, Writing – review & editing. RP: Formal analysis, Investigation, Methodology, Software, Validation, Writing – review & editing. ArC: Conceptualization, Investigation, Writing – review & editing. MT: Conceptualization, Investigation, Writing – review & editing. AZ: Investigation, Writing – review & editing. SB: Data curation, Software, Writing – review & editing. AlC: Data curation, Software, Writing – review & editing. AP: Investigation, Writing – review & editing. ID: Data curation, Software, Validation, Writing – review & editing. CH: Funding acquisition, Methodology, Resources, Software, Supervision, Writing – review & editing. CP: Conceptualization, Funding acquisition, Methodology, Supervision, Writing – review & editing. GV: Funding acquisition, Supervision, Writing – review & editing. MM: Conceptualization, Funding acquisition, Resources, Supervision, Writing – review & editing. CM: Conceptualization, Data curation, Formal analysis, Funding acquisition, Investigation, Methodology, Resources, Software, Supervision, Validation, Visualization, Writing – original draft.
