# Supplementary material for: On the effects of 30.5 GHz sinusoidal wave exposure on glioblastoma organoids
Source: Front Oncol. 2024 May 31;14:1307516. doi: 10.3389/fonc.2024.1307516 (PMC11176452; doi:10.3389/fonc.2024.1307516)
Supplement: Supplementary file 1 [file DataSheet_1.docx]

Supplementary Material

# Supplementary Figures and Tables

## Supplementary Figures


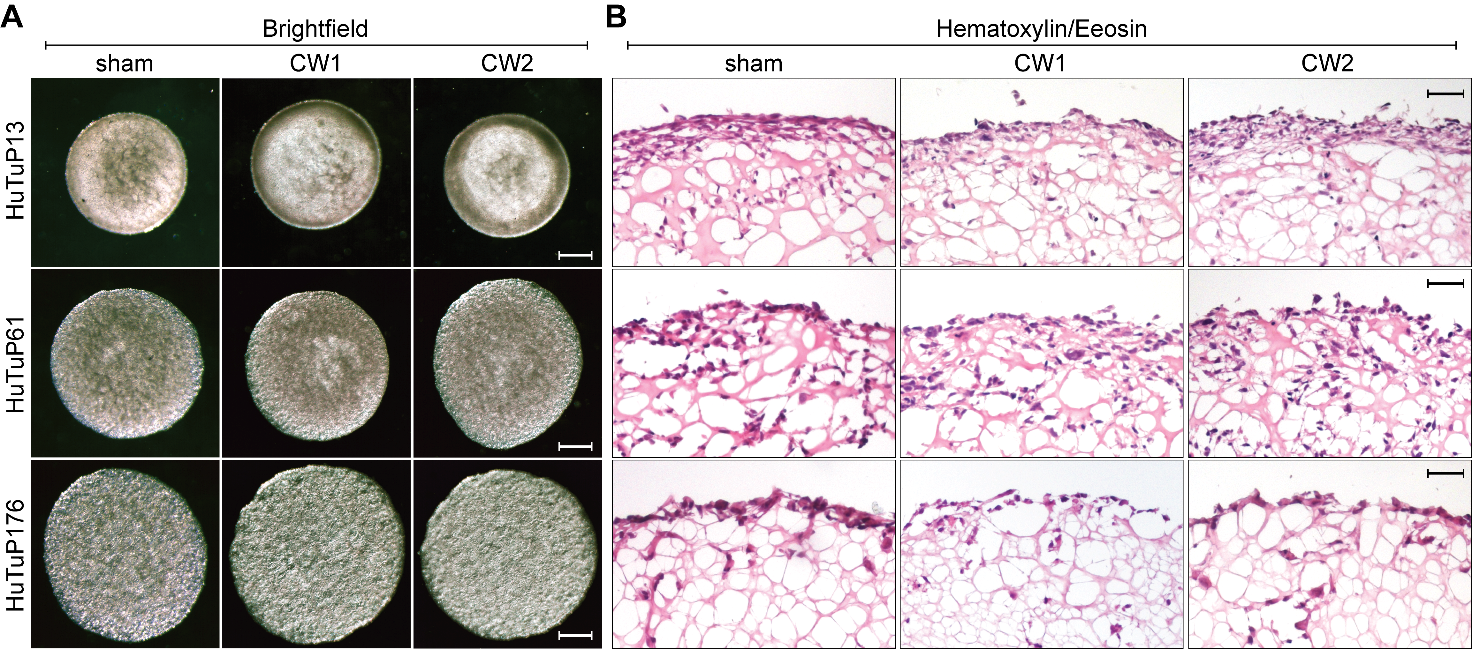


**Supplementary Figure S1.** Suppl. Fig. 1: (A) Representative bright field images of GBM organoids, generated with HuTuP13, 61, and 176 primary cells, after 72 hrs from being exposed to sham, CW1, or CW2 protocols. Original magnification: 3.2x; bar: 500µm. (B) Representative Hematoxylin and Eosin staining of GBM organoids treated as in (A). Original magnification: 10x; bar: 50µm.


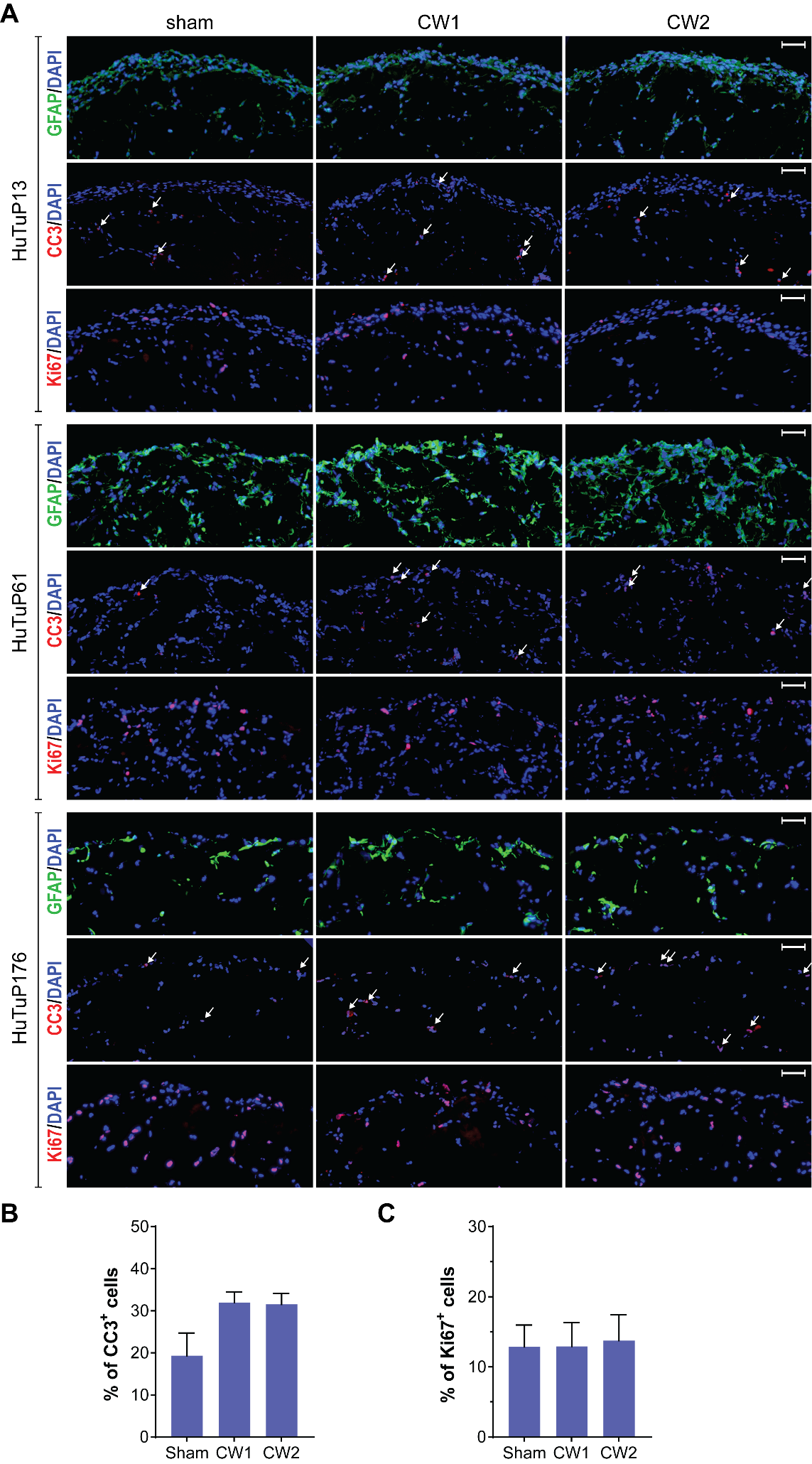


**Supplementary Figure S2.** (A) Immunofluorescence analysis displaying representative control and CW-treated (24 hrs) GBM organoids (HuTuP13, HuTuP61, and HuTuP176) stained for GFAP (green, upper panels), Cleaved Caspase 3 (CC3; red, middle panels), and Ki67 (red, lower panels). Original magnification: 10x; bar: 50µm. (B, C) Relative quantifications of CC3^+^ (B) and Ki67^+^ (C) cells in HuTuP13, 61, and 176 GBM organoids treated as indicated for 24hrs.


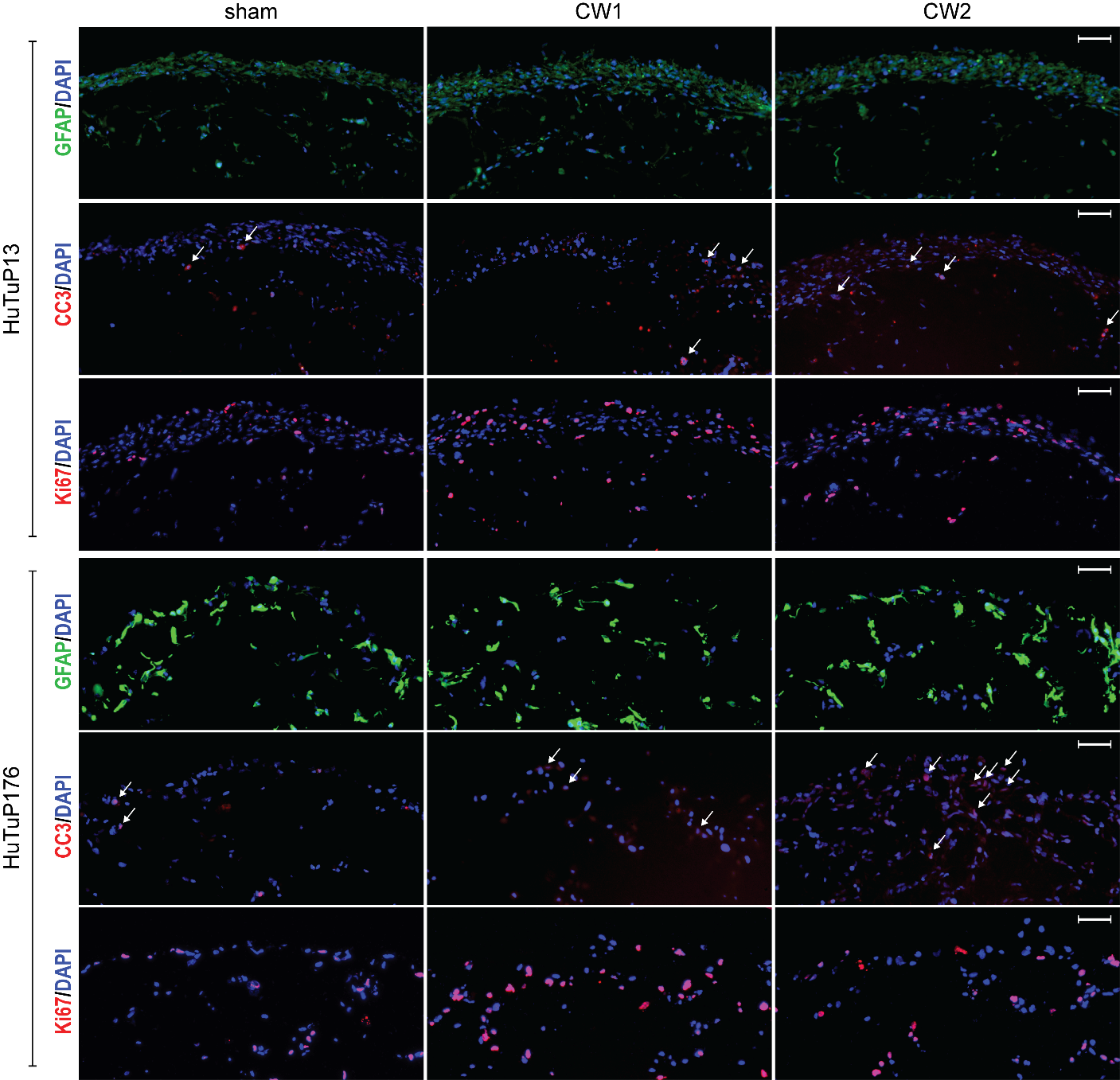


**Supplementary Figure 3.** Immunofluorescence analysis displaying representative control and CW-treated (72 hrs) GBM organoids (HuTuP13 and HuTuP176) stained for GFAP (green, upper panels), Cleaved Caspase 3 (CC3; red, middle panels), and Ki67 (red, lower panels). Original magnification: 10x; bar: 50µm.

**Supplementary Table 1**: Primer sequences of the relevant genes analyzed by RQ-PCR.

| **FEN1_for** | ACCAAGCTTTAGCCGCCGAG |
| --- | --- |
| **FEN1_rev** | CTGGGGGCCACATCAGCAAT |
| **RAD50_for** | CAATGGGGCGGGAAAGACGA |
| **RAD50_rev** | GCAGACGAATCTGGGCTCTCA |
| **RAD51_for** | CGCTGCGGACCGAGTAATGG |
| **RAD51_rev** | CGCATAGGCAACAGCCTCCA |
| **ATR_for** | GGCATTCCAAAGCGCCACT |
| **ATR_rev** | AACGGCAGTCCTGTCACTCTAT |
| **BRCA2_for** | GCAGCAGACCCAGCTTACCT |
| **BRCA2_rev** | CGCAACTTCCACACGGTTGT |
| **BRCA1_for** | TCTGAGGACAAAGCAGCGGA |
| **BRCA1_rev** | CCCTGGTTCCTTGAGGGGTG |
| **EXO1_for** | GCGGGAATTGTGCAAGCCAT |
| **EXO1_rev** | TGCACATTCCTAGCCGAGCTT |
| **BARD1_for** | AGCCTGTGTGTTTAGGAGGATGTG |
| **BARD1_rev** | TGTATCCAGGCCGGGGTGTAA |
